# The Public's Medical Guides

**Published:** 1912-11-02

**Authors:** 


### THE PUBLIC'S MEDICAL GUIDES.

There are many ways of setting forth medical or semi-medical lore for popular consumption. At one end of the scale come such refined and artistic products as *Confessio Medici* and *The Corner of Harley Street*, to name two fairly recent specimens of the very highest order of merit. At the other are the prescribing columns of a certain sort of Sunday newspaper, the *Family Doctor*, and similar effusions. Between stand a miscellaneous collection, many of them more or less praiseworthy and beneficial to those who read them. One such type is that of the every-man-his-own-doctor book, a form of publication which has its own distinct advantages and disadvantages.

A generation ago the once famous *Dictionary of Medicine*, edited by the late Sir Richard Quain, was to be found in very many non-medical households. Although not expressly designed for lay readers, it yet possessed distinct merits from that point of view. The scope was comprehensive, the articles were not too long or too technical, and the state of medical science at that day rendered it much easier than it is now for an intelligent layman to comprehend the instruction offered in a medical text-book. It was a thoroughly sound book of its date, although the prevention of disease as distinct from its cure was hardly even thought of by the editor and his contributors. The *Dictionary* has been brought up to date from time to time, but the present generation (both of the medical profession and of the public) does not reverence it as did that of the 'eighties. Indeed, it is not easy to say what is now the best-selling medical manual among the general public. Possibly the very latest * may achieve that distinction in the near future.

Dr. Grimshaw is a great reader of poetry, and there is hardly a disease concerning which he cannot print some tag or other, as often as not somewhat inapposite. This, we submit, is a waste of time and space in a medical guide, in which a strict attention to business should be the keynote. The dogmatic note struck is, of course, the right one for a popular work, but it is struck a little too hard, and a strain of egotism is intermingled with it.

Let us turn, however, to the author's strong points, which are many. First and foremost he emphasises everywhere the prevention and avoidance of disease and the main principles of modern hygiene. In simple language, which it will tax the most misguided stupidity to misunderstand, he shows how and why infection and disease are spread; this is perhaps the most outstanding and most valuable feature of the book. Next he gives comparatively few prescriptions for diseases, preferring to indicate the general outlines of proper treatment without attempting to supersede the functions of the medical attendant. (By a printer's error on p. 561 strychnine is prescribed in a dose of one grain.) He is not afraid or ashamed to say that the cause of some particular disease is unknown, or that its treatment is little likely to be successful. He is especially caustic in exposing the subterfuges which are almost forced upon the profession by the inability of the public to appreciate a suspended judgment or a prudent hesitation in diagnosis; such subterfuges, for example, as the common idiom that a patient is "threatened with consumption," or that "congestion of the lungs" has "turned to pneumonia." Yet another of Dr. Grimshaw's virtues is his fearless exposure of quack methods and quack medicines. At the end of several of the sections he quotes from the British Medical Association's analyses of patent medicines the composition of those especially vaunted as infallible cures, exposing on the familiar lines the gross disproportion between the high prices for which these secret and proprietary nostrums are vended and the almost infinitesimal cost of their ingredients. He is fully up to date and abreast of the latest advances in radiotherapy, organotherapy, and has produced a "popular medical guide" worthy of the name, and reasonable in price

* *The People's Medical Guide.* By John Grimshaw, M.D. J. and A. Churchill. 8s. 6d. net.

